# Perspective on Perinatal Birth Canal Injuries: An Analysis of Risk Factors, Injury Mechanisms, Treatment Methods, and Patients’ Quality of Life: A Literature Review

**DOI:** 10.3390/jcm14103583

**Published:** 2025-05-20

**Authors:** Patrycja Głoćko, Sylwia Janczak, Agnieszka Nowosielska-Ogórek, Wiktoria Patora, Olga Wielgoszewska, Mateusz Kozłowski, Aneta Cymbaluk-Płoska

**Affiliations:** Department of Reconstructive Surgery and Gynecological Oncology, Pomeranian Medical University in Szczecin, Al. Powstańców Wielkopolskich 72, 70-111 Szczecin, Poland; patrycjaglocko@gmail.com (P.G.); anowosielska14@gmail.com (A.N.-O.); wiktoria19909@gmail.com (W.P.); olgawielgoszewska@gmail.com (O.W.); mateusz.kozlowski@pum.edu.pl (M.K.); aneta.cymbaluk@gmail.com (A.C.-P.)

**Keywords:** perineal trauma, obstetric injuries, OASIs, episiotomy, vaginal delivery, maternity care, birth trauma, medical ethics

## Abstract

Perineal injuries are a common complication of vaginal delivery, affecting 75–85% of women. This review examines current knowledge on risk factors, classification, treatment, and quality of life impacts. Risk factors are divided into maternal, foetal, and labour-related categories. Treatment depends on injury severity. First-degree tears can be managed conservatively, with skin glue or suturing—preferably with synthetic absorbable sutures to reduce pain and infection risk. Second-degree tears and episiotomies respond best to continuous non-locking sutures, improving healing, and minimizing postpartum pain. Severe third- and fourth-degree tears require specialised surgical techniques, such as the overlay method for anal sphincter repair, which improves faecal continence. Proper preoperative care, including antibiotics and anaesthesia, enhances outcomes. Episiotomy is controversial; selective use based on clinical indications is recommended over routine practice. Research shows no significant long-term benefits compared to spontaneous tears, and links episiotomy to psychological distress and negative body image. Preventative strategies, like perineal massage and warm compresses during labour, may reduce the risk of severe trauma, particularly in first-time mothers. Perineal trauma can have lasting physical and psychological effects, impacting sexual function, continence, and mental health. Proper diagnosis, treatment, and postpartum care are essential. Future studies should aim to standardise care protocols and explore long-term outcomes to enhance patient quality of life.

## 1. Introduction

The female perineum is a fibromuscular region located between the anus and the posterior limit of the vulvar orifice, comprising the perineal body—where several muscles and fascial layers converge—and forming part of a rhomboid-shaped area that supports urogenital and anorectal functions [1]. Millions of women worldwide have perineal damage during childbirth each year. More than 85% of women having a vaginal birth suffer some perineal trauma [2]. The reported incidence of perineal trauma in primiparous women ranges from 5.1 to 8.3% for third- and fourth-degree tears involving obstetric anal sphincter injuries (OASIs) and from 35.1 to 78.3% for second-degree tears. The data for multiparous women are as follows: the incidence of second-degree tears is 34.8–39.6%, while third- and fourth-degree tears occur in 1.8–2.8% of cases [3]. However, severe injuries that could cause future dysfunctions are less common, occurring between 6.4% and 11% of the time [4]. There are many risk factors that may contribute to an increased frequency of perineal injuries, and they should be taken into consideration in the care of pregnant and labouring women. Mothers who have perineal injuries may experience both immediate and long-term difficulties. Infection and dehiscence are possible short-term consequences. Long-term issues include dyspareunia, discomfort, chronic pelvic pain, faecal and urine incontinence, pelvic organ prolapse, anal sphincter injuries, and psychological issues like relationships between mothers and their children and a partner are also possible for some. All those consequences can influence the relationship between the mother and baby, intimate relations, breastfeeding, and the sense of postpartum recovery [5,6,7]. According to the Royal College of Obstetricians and Gynaecologists (RCOG), there are 4 degrees of perineal damage. Only the vaginal mucosa and perineal skin were affected in the first degree of trauma; the perineal musculature was affected in the second degree; the anal sphincter was affected in the third degree; and the rectal mucosa was affected in the fourth degree [8].

The objective of this literature review is to provide a comprehensive and multidimensional overview of perinatal genital tract injuries, including their anatomical and physiological background, risk factors, classification, treatment options and their effectiveness—both conservative and surgical—as well as modern therapeutic approaches, postoperative care, and the long-term impact on women’s physical and mental health, including quality of life.

## 2. Methods

To develop this study, a review of scientific literature concerning obstetric perineal injuries was conducted. The selection of materials was based on a search of reputable scientific databases: PubMed and Google Scholar.

The search covered publications from 2015 to 2025 in order to ensure the relevance and currency of the gathered data. Keywords used in the search included the following: “perineal trauma”, ”obstetric injuries”, “OASIS”, “episiotomy”, “vaginal delivery” and “risk factors for perineal injury”, “maternity care”, “birth trauma”, “medical ethics”.

Inclusion criteria were as follows:

- Publications in English.
- Studies involving women giving birth vaginally.
- Papers discussing risk factors, prevention, classification, and consequences of perineal trauma related to childbirth.

The following types of publications were excluded:

- Articles lacking empirical data (e.g., editorials, letters to the editor).
- Studies focused exclusively on perineal injuries not related to childbirth.
- Publications prior to 2015.

The selected articles were assessed for methodological quality and relevance to the research topic. Based on this analysis, a synthesis of current knowledge was developed, along with the identification of research gaps concerning childbirth-related perineal injuries.

**Figure 1:**
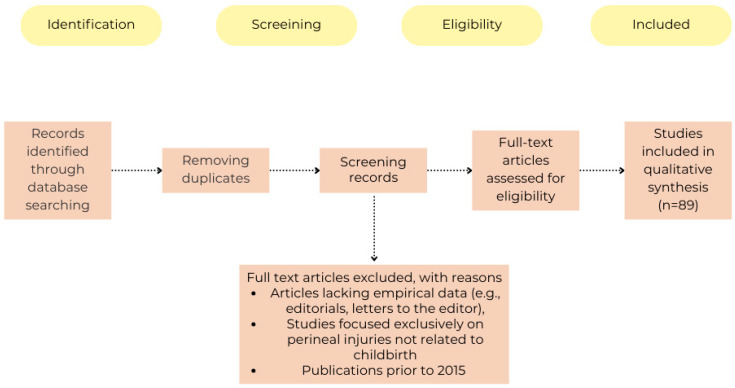
Flow chart.

Figure 1 visually represents the selection process of the included studies, from initial identification to final inclusion.

## 3. Anatomy and Physiology of Childbirth

Childbirth is a physiological process in which the foetus and placenta are expelled from the uterus [9]. International literature identifies three main stages of labour [9]:

1. First stage:
  ○ Latent phase: Lasting approximately 8 h, during which uterine contractions are irregular, and the cervix is not yet dilated.
  ○ Active phase: Characterised by stronger and more regular contractions leading to cervical dilation. This stage ends when the cervix is fully dilated to 10 cm [9].
2. Second stage: Begins with full cervical dilation. During this stage, the foetus descends into the birth canal, assisted by the mother’s efforts. For first-time mothers (primiparas), this stage usually lasts up to 3 h, while for women with previous deliveries (multiparas), it lasts less than 2 h. Exceeding these times is considered prolonged. This stage ends with the birth of the baby [9].
3. Third stage: Covers the time from the baby’s birth to the expulsion of the placenta. It typically lasts 5 to 30 min; delays beyond 30 min increase the risk of postpartum haemorrhage and may require medical intervention [9].

## 4. Risk Factors

Risk factors for perineal trauma can be divided into three categories: maternal, foetal, and labour-related (Table 1).

### 4.1. Maternal Risk Factors

Maternal factors that may contribute to the perineal trauma (especially those that increase the risk of third- or fourth-degree perineal tears) are maternal age above 25 years, abnormal collagen production, inadequate nutritional state, and higher socio-economic background [2,10]. While Bączek et al. proved that advanced maternal age has been associated with an increased risk, while research (Nolan et al. 2021) indicates that maternal age over 35 may have a protective effect against obstetric anal sphincter injuries (OASIs) with a lower incidence in this age group (1.3% vs. 1.9%) [11,12]. Women who sustained OASIs were found to deliver at a slightly later gestational age (39.8 vs. 39.5 weeks, *p* < 0.001) and were significantly younger (29.4 vs. 30.4 years, *p* < 0.001) compared to those with an intact sphincter. These findings suggest that maternal age should be carefully considered when assessing perineal trauma risk [12]. Ethnicity, particularly Asian descent, is considered a risk factor [2,13,14]. Notably, Black and Latina women exhibited a decreasing incidence of lacerations with increasing severity, in contrast to the upward trend observed among Asian and White women. While this finding highlights a potential racial and ethnic disparity, the underlying causes remain unclear [15]. It is important to note that other contributing factors, such as foetal weight and head size, may also contribute to a higher incidence of severe perineal lacerations in women from certain ethnic groups. Women attempting their first vaginal birth at all and first vaginal birth after caesarean (VBAC) face a notably higher risk of experiencing anal sphincter injuries [16]. Perineal length plays an important role in the development of perineal trauma. Patients with a perineal body of 3 cm or less were significantly more likely to experience third- or fourth-degree laceration [17]. We should also consider the impact of the labouring woman’s BMI. Increased BMI or excessive weight gain during pregnancy does not influence the risk of genital tract trauma at birth; however, higher BMI at booking is associated with a reduced incidence of minor perineal trauma while showing no correlation with the occurrence of OASIs (obstetric anal sphincter injuries) [18]. This issue requires further research.

### 4.2. Foetal Risk Factors

Predisposing foetal factors for perineal trauma include, among others, a birth weight greater than 4 kg [10]. A study examining the incidence of perineal trauma found rates of 7% in the occipito-posterior position compared to 1% in the occipito-anterior position, with the difference being highly significant (*p* < 0.001) [19]. The occipito-posterior position is less physiological than the occipito-anterior position, which increases the strain on perineal tissues and raises the risk of injury. We also need to mention another risk factor which is shoulder dystocia. Shoulder dystocia occurs when the foetus’s anterior, posterior, or both shoulders are not delivered during childbirth. It happens when one of the foetus’s shoulders is unable to slip beneath the mother’s pubic symphysis. Internal rotation and foetal descent are delayed as a result [20]. To effectively manage this situation, it is important to use some of manoeuvres (McRoberts manoeuvre, suprapubic pressure, Rubin II, Wood’s, reverse Woods, Gaskin all-fours manoeuvre, deliver posterior arm or shoulder, abdominal rescue, Zavanelli manoeuvre, clavicular fracture) [21]. Woods’ screw and reverse Woods’ screw manoeuvres were found to be independently linked to an increased incidence of OASIs [20].

### 4.3. Labour-Related Risk Factors

The presence and degree of perineal damage at the initial birth enhance the chance of spontaneous tears at subsequent deliveries [22]. There is a strong association between instrumental delivery (forceps, vacuum), in particular without episiotomy. If a normal vaginal birth is compared to a vacuum birth, then vacuum is associated with a higher number of maternal complications, the most common of which are perineal tears [10,23]. The same is true when it comes to using forceps (it increases the risk of perineal tears; the risk of a tear is six times higher with a forceps delivery without an episiotomy than with a vaginal birth without one) [10]. If these two instrumental methods (vacuum and forceps) are compared with each other, then the conclusions are as follows: using forceps increases the probability of suffering a perineal injury more than using ventouse [10,24].

An episiotomy is a surgical cut made in the vaginal opening and perineum to facilitate the infant’s head passing through during the crowning phase of vaginal childbirth. When it comes to episiotomy, several aspects should be considered. There is restrictive and routine episiotomy. Compared with routine use, restrictive episiotomy resulted in less severe perineal trauma, less suturing and less healing time [25]. Current studies revealed that midline episiotomy links to risk of birth trauma during delivery in comparison to mediolateral episiotomy [26]. According to available data, an episiotomy suture angle of 40–60° seems to fall within the safe range [14].

The findings revealed that episiotomy reduced the risk of severe perineal lacerations during forceps-assisted deliveries but showed no protective effect in spontaneous vaginal births or vacuum-assisted deliveries [27]. There are studies that prove that lateral or mediolateral episiotomy reduces the incidence of OASIs in operative vaginal delivery, particularly in nulliparous ventouse or forceps deliveries [28,29].

Kapoor D.S. et al. found that episiotomy scars with a depth greater than 16 mm, a length exceeding 17 mm, an incision point more than 9 mm lateral to the midpoint, and an angle between 30° and 60° are significantly linked to a reduced risk of OASIs [14]. Surgical incision of the perineum is linked to heightened postoperative discomfort and early postpartum dyspareunia [30].

Higher rates of trauma have been linked to standing upright or the lithotomy position with stirrups, but lower rates have been identified for women who choose semi-sitting, lateral, or squatting positions [31]. The risk of severe perineal laceration increases with duration until the third hour of second stage of labour. Women with a second stage of labour lasting more than two hours were at higher risk than those with a second stage lasting one hour or less [32].

The use of oxytocic augmentation during labour has been shown to be significantly associated with severe perineal trauma [19]. It is worth mentioning the impact of epidural analgesia use on the incidence of perineal traumas. In this regard, Baczek et al. in their 2022 retrospective analysis, demonstrated that epidural analgesia was associated with an increased risk of perineal trauma, including a twofold increase in the risk of perineal laceration and an over fivefold increase in the risk of episiotomy [33].

**Table 1:** Risk factors for perineal trauma during childbirth.

Category	Risk Factors	Key Factors	Reference
Maternal Factors	Advanced maternal age	Traditionally associated with an increased risk of perineal trauma [11]. However, some studies suggest that maternal age over 35 may have a protective effect against obstetric anal sphincter injuries (OASIs) [12].	[11,12]
	Abnormal collagen production	Potentially weakens perineal tissue, increasing risk.	[2]
	Inadequate nutritional state	May affect tissue integrity and healing.	[2]
	Higher socio-economic background	Linked to an increased risk of severe perineal trauma.	[2]
	Ethnicity	Asian descent is a risk factor [2,10,15] while Black and Latina women have a lower rate of perineal laceration compared to White women [15].	[2,10,15]
	First vaginal birth and VBAC	Increased risk of OASI.	[16]
	Perineal length	A perineal body of 3 cm or less is significantly associated with third- or fourth-degree lacerations.	[17]
	BMI	Increased BMI does not influence genital tract trauma risk but is linked to a reduced incidence of minor perineal trauma; no correlation with OASIs.	[18]
	Gestational age	Women with OASIs delivered at a slightly later gestation. However, gestational age was not a significant risk factor in regression models.	[12]
Foetal Factors	Birth weight > 3 kg	Associated with a higher risk of perineal trauma.	[10]
	Occipito-posterior position	Increased perineal trauma.	[19]
	Shoulder dystocia	Causes delayed foetal descent and internal rotation, leading to increased perineal trauma. Woods’ screw and reverse Woods’ screw manoeuvres are linked to a higher incidence of OASIs.	[20]
Labour-Related Factors	Previous perineal damage	Higher risk of perineal tears in subsequent deliveries.	[22]
	Instrumental delivery	Higher risk of perineal tears with forceps than vacuum.	[10,24]
	Episiotomy	Restrictive episiotomy leads to less severe perineal trauma than routine use [25]. Midline episiotomy increases the risk of birth trauma. Lateral/mediolateral episiotomy reduces OASI risk in operative vaginal delivery [26].	[25,26]
	Episiotomy technique	A suture angle of 40–60° reduces OASI risk. Incisions deeper than 16 mm, longer than 17 mm, and more than 9 mm lateral to the midpoint are protective.	[14]
	Maternal position	Higher trauma rates in lithotomy/stirrups positions; lower rates in semi-sitting, lateral, or squatting positions.	[31]
	Prolonged second stage of labour	Risk increases after 2 h, significantly higher after 3 h.	[32]
	Oxytocin augmentation	Significantly associated with severe perineal trauma.	[19]
	Epidural analgesia	Associated with an increased risk of perineal laceration.	[33]

## 5. Perineal Injuries

The external female genitalia consist of the labia majora and minora, clitoris, mons pubis, vaginal vestibule, and perineal body. The perineal body, located between the anus and the vaginal vestibule, can be injured during childbirth, as can the labia, cervix, vagina, and anal sphincter [26]. Perineal injuries occur in 75–85% of women during spontaneous vaginal deliveries [34]. Although many of these tears heal without long-term consequences [26], more severe cases, depending on their extent, can lead to postpartum complications, including increased pelvic floor muscle dysfunction, as well as chronic pain, bleeding, infections, dyspareunia, and urinary or rectal fistulas [34].

The Royal College of Obstetricians and Gynaecologists (RCOG) and the International Consultation on Incontinence have endorsed the following classification of perineal injuries developed by Sultan [8]:

- First-degree tear: Superficial injury to the vaginal mucosa, which may also involve the perineal skin without affecting pelvic floor muscles.
- Second-degree tear: Injury characteristic of first-degree tear but extending to the perineal muscles.
- Third-degree tear: A second-degree tear with additional injury to the anal sphincter complex, further divided into three subcategories:
  ○ Grade 3a—injury to less than 50% of the external anal sphincter,
  ○ Grade 3b—injury to more than 50% of the external anal sphincter,
  ○ Grade 3c—complete rupture of both the external and internal anal sphincters.
- Fourth-degree tear: Tear involving the anal sphincter and the anorectal mucosa [8]. The National Institute for Health and Care Excellence (NICE) recommends suturing first-degree tears to prevent wound dehiscence and promote proper healing unless the wound edges are naturally well aligned [35]. However, surgical suturing may lead to increased sensitivity and localised pain. To mitigate these issues, some studies propose the use of surgical glue, while others suggest refraining from surgical intervention altogether for such tears [36]. Surgical glue has been recognised as an effective method for repairing Grade I perineal tears in physiological deliveries, reducing procedure time, garnering higher patient satisfaction, and providing safe and aesthetically favourable outcomes [37,38].

The repair of second-degree tears involves approximating the torn tissues [39]; however, these injuries can be highly complex, often leading to misclassification and improper treatment by medical personnel [40]. Consequently, challenges such as suture healing problems, pain, delayed return to sexual activity, or haemorrhoids may arise, significantly affecting a woman’s quality of life during this period [41]. To address these challenges, Sweden introduced a new classification system for Grade II injuries, accounting for the diversity of perineal tears by dividing them into subcategories:

- 2a: damage involving less than 50% of the perineal muscle,
- 2b: damage involving more than 50% of the perineal muscle,
- 2c: damage involving the entire perineal muscle [42].

Studies indicate that patients with 2C injuries reported significantly higher pain scores compared to women with 2A and 2B tears [43], emphasizing the need for more precise classification of moderate injuries.

Obstetric anal sphincter injuries (OASIs), classified as third- and fourth-degree tears, are among the most severe complications of vaginal deliveries [44]. Reported incidence rates of OASIs range from 4% to 11% in women delivering vaginally [45]. Diagnosing OASIs requires a thorough perineal examination, including the assessment of all tissue layers and a rectal examination, which is crucial for diagnosis [46]. If doubts arise, consultation with a second specialist is recommended to improve diagnostic accuracy. The use of ultrasound during childbirth can further aid in detecting OASIs, enhancing the identification of these complications [46].

The use of evidence-based techniques to achieve an adequate primary repair is crucial for reducing the risk of wound infection, breakdown, or incomplete healing of the anal sphincter complex [47]. Moreover, the application of proper repair techniques can prevent long-term complications like pain, dyspareunia, and faecal incontinence [47]. Since the primary procedure offers the highest chance of success [48], reconstruction can safely be delayed by 8–12 h to involve a more experienced specialist [49]. Effective surgery requires proper lighting and visibility, appropriate surgical instruments and suture material, as well as adequate anaesthesia [39].

## 6. Childbirth-Related Complications

Childbirth-related complications may affect the pelvic floor, reproductive organs, or mental health. To enhance clarity and reduce redundancy, the following table outlines key postpartum and obstetric complications as distinct entities. Each condition is presented with its clinical characteristics, risk factors, diagnostic strategies and symptoms (Table 2).

## 7. Pathophysiology of Injuries

Peripartum injuries to maternal tissues include both superficial injuries and more severe ones, such as uterine rupture or anal sphincter damage [8,58]. The mechanisms behind these injuries can be explained by the interaction of physiological, biomechanical, and anatomical factors influencing the course of labour [69]. Childbirth involves significant physiological changes in the mother’s body that enable the foetus to pass through the birth canal [70]. The mechanical forces during labour can exceed the elastic limits of the perineal tissues, vagina, pelvic floor muscles, and supporting structures, especially in cases of a large foetus, improper foetal positioning, or rapid labour. As a result, tears or ruptures in these tissues may occur [70,71]. It has also been found that the risk of severe tears (third- and fourth-degree tears according to Sultan’s classification) of the perineum increases when the second stage of labour is prolonged beyond 3 h. Instrumental delivery is the greatest risk factor [72], particularly when combined with a large foetus (large head circumference, high birth weight) or occipito-posterior foetal position. A prolonged second stage of labour is also associated with adverse maternal outcomes such as postpartum haemorrhage, fever, infections, and urinary retention [32]. The relationship between the length of the second stage and OASIs (obstetric anal sphincter injuries) is influenced by the method of delivery and the duration of labour, though maternal age may also contribute to the risk [73].

## 8. Methods of Treating Perinatal Genital Tract Injuries

Treatment of perinatal genital tract injuries depends on the degree and type of damage, and the choice of method is based on the latest guidelines, current research, and the capabilities of the therapeutic team, including the patient. It is a complex process that requires an individual approach, encompassing a wide range of therapeutic methods. Modern medicine offers a choice of both conservative and surgical treatment, which can be further enriched with innovative treatment methods. One of the elements is also postoperative care, which influences the essence of building therapeutic success [39,74].

### 8.1. Conservative Treatment and Prevention

In the case of minor injuries, such as a superficial perineal laceration, conservative treatment is mainly used, focusing on pelvic floor muscle rehabilitation. Regular exercise, individually selected to the patient’s capabilities, allows for the strengthening of the pelvic floor muscles, which translates into better control of bladder and sphincter function [24]. Biofeedback is used to monitor and observe progress, providing the patient with feedback on pelvic floor muscle activity [2]. In patients in whom independent exercise is not possible, electrostimulation may be helpful, stimulating the pelvic floor muscles with low-intensity current impulses [39].

### 8.2. Surgical Techniques

Injuries such as a third- or fourth-degree perineal laceration according to the Sultan scale and anal sphincter injury require surgical intervention [24]. Modern techniques allow for accurate reconstruction of damaged tissues and minimise the risk of complications.

To restore proper function and structure in the case of perineal wounds, a layered stitching technique can be used, consisting of restoring the continuity of each layer (vaginal mucosa, fascial tissue, and muscle tissue) [74]. The choice of sutures and technique depend on the exact location, extent, and depth.

When the anal sphincters are damaged, the preferred procedure is sphincteroplasty, the aim of which is to restore the continuity of the sphincters using the “end-to-end” or “overlap” method [39].

To minimise the risk of extensive perineal injuries, an episiotomy is performed, which can be performed in the midline (midline episiotomy) or obliquely (mediolateral episiotomy) [74].

### 8.3. Modern Technologies and Therapies

Modern medicine offers modern technologies and therapies to maximise treatment outcomes. Among them, we can distinguish transvaginal ultrasound, which is a tool that facilitates the assessment of the degree of tissue damage, allowing for additional assessment of the healing process [75]. It is also used to assess the function of the anal sphincters and pelvic floor muscles.

Laser therapy is a technique used to accelerate wound healing due to improved blood supply to the area where the laser was applied [76]. It also helps in reducing pain and can be combined with other treatment methods, e.g., physiotherapy.

The regenerative potential of stem cells is also used in the treatment of perinatal injuries; although it is still in the clinical trial phase, preliminary results indicate significant effectiveness in rebuilding damaged structures [77].

### 8.4. Postoperative Care

Postoperative care is as important as the procedure itself in terms of the patient’s return to full health. The prescription of painkillers, proper education, including proper postoperative wound hygiene, pain control, or how to recognise disturbing symptoms of infection, as well as early initiation of physiotherapy have a significant impact on improving the patient’s health and the outcome of surgical treatment [39]. In the case of trauma related to the occurrence of injury, it is worth securing the patient in terms of psychological care. A holistic approach to the patient will allow for the best results.

## 9. Quality of Life Assessment

**Figure 2:**
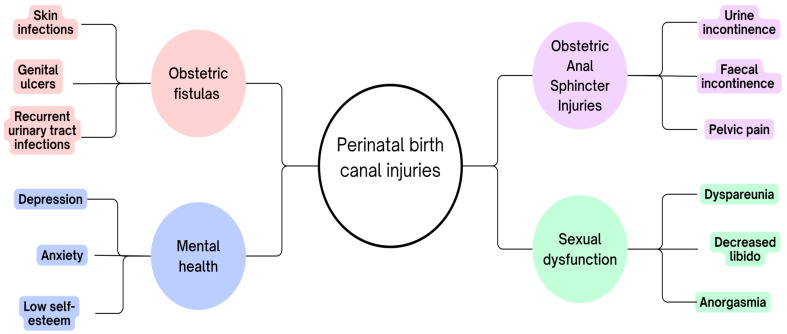
Adverse consequences of perinatal injuries.

Perineal injuries are a common consequence of vaginal delivery, affecting 50% to 90% of women, with 4% to 11% experiencing obstetric anal sphincter injury (OASI) [74]. These injuries, particularly OASIs, are associated with both short- and long-term consequences (Figure 2). Long-term effects include perineal pain, dyspareunia, delayed return to sexual activity, which, over time, can lead to depression [78]. A correlation has been observed between perineal trauma and the occurrence of physical symptoms: women who experienced such trauma were more likely to report various physical complaints. Moreover, a greater number of reported physical symptoms was associated with a higher risk of postpartum depression, anxiety, and post-traumatic stress (PTS) symptoms. Based on this, it can be suggested that physical ailments during the postpartum period may play a significant role in the development of negative psychological consequences after childbirth [5]. As O’Shea et al. present, almost 47% of women were found to have female sexual dysfunction based on the Female Sexual Functioning Index (FSFI). Additionally, 34% of women had the Golombok Rust Inventory of Sexual Satisfaction (GRISS) score of five or higher, which is indicative of sexual dysfunction [79]. The postpartum period is not only mentally but also physically demanding, and the added impact of perineal injuries significantly aggravates the challenges faced by a new mother in adapting to her new circumstances [74]. The most common complications of OASIs are urine incontinence, with faecal incontinence being less frequent. Such issues significantly deteriorate the quality of life for postpartum women, often leading them to avoid social interactions and feel embarrassed in public spaces and causing low self-esteem [80]. Furthermore, pelvic floor disorders, including urinary incontinence, anal incontinence, pelvic organ prolapse, and pelvic pain, can significantly hinder a woman’s motivation and capacity for physical activity, posing a broader risk to overall health and well-being [81].

## 10. Effectiveness of Treatment

Depending on the degree of perineal injury, different techniques and procedures are employed. For the first-degree tears, conservative methods, the use of a skin glue, or surgical suturing can be considered [74]. When using a skin glue, the procedure is shorter and less painful, and the cosmetic and functional outcomes are not inferior to traditional suturing. This was confirmed in a prospective, randomised controlled trial (RCT) with a non-inferiority design, comparing the use of skin glue to traditional suturing in the treatment of the first-degree perineal tears [39,74].

Regarding the types of sutures used, it has been shown that synthetic absorbable sutures are associated with less short-term pain and, consequently, a reduced need for analgesics compared to catgut sutures [39]. No significant differences were observed in short-term or long-term pain levels, nor in the wound healing process, between the use of standard multifilament sutures, rapidly absorbing multifilament sutures, and monofilament sutures [74]. However, due to their less complex surface, monofilament sutures have been shown to exhibit a lower presence of bacteria, which contributes to a reduced risk of infection [82].

The repair of the second-degree tears and episiotomies should include the closure of layers—vaginal epithelium, perineal muscles, rectovaginal fascia, and skin—with a preference for continuous non-locking sutures. These techniques reduce pain, swelling, and the risk of tissue damage [50]. A Cochrane review encompassing 16 studies demonstrated that continuous techniques are associated with less postpartum pain, and RCTs have shown that continuous sutures result in better sexual function and shorter procedure times compared to interrupted sutures [74]. The third- and fourth-degree tears are more complicated, which is why clinical experience and appropriate techniques are recommended to achieve satisfactory treatment outcomes. An important aspect is the preparation of the patient before the procedure, which includes the administration of antibiotics that significantly reduce the risk of wound complications, as well as proper anaesthesia, preferably regional or general, and a lithotomy position with limbs supported [8]. Operating room conditions are also crucial, and proper lighting and visibility are recommended [8]. In the case of OASI tears, the restoration of the anal sphincter complex and the rectal mucosa is essential, depending on the extent of the injury. For repairing the rectal mucosa, a continuous monofilament suture is recommended, as it reduces the risk of infection. When repairing the external anal sphincter, the preferred technique is the overlay technique, which, according to a 2013 Cochrane review, provides better short-term outcomes, such as less faecal urgency and anal incontinence symptoms at 12 months [47]. Other damaged layers are repaired similarly to a second-degree tear, using continuous sutures, which ensure better tissue approximation, a shorter repair time, and a lower risk of complications [74].

The practice of episiotomy has been the subject of much debate regarding its relevance in perineal injuries. Over time, indications for performing episiotomy as a preventive measure to avoid further perineal damage have evolved. Therefore, limited use of episiotomy is recommended, guided by specific clinical indications, rather than adopting it as a routine procedure [50]. Controlled studies examining long-term outcomes, such as urinary and faecal incontinence and dyspareunia, have shown no significant differences between spontaneous perineal tears during childbirth and episiotomy [50]. Interesting findings were presented in cohort studies that evaluated body image, perception of childbirth as traumatic, psychological stress, perineal pain, impact on parenting tasks, and the mother–infant bond at 6–12 weeks (n = 103) and 6–10 months postpartum (n = 91). At 6–12 weeks, women who underwent episiotomy were more likely to have negative feelings about their appearance compared to women with OASIs. Both women with OASIs and those who underwent episiotomy were more likely to perceive childbirth as a traumatic experience, with women with OASIs reporting more symptoms related to this perception [83]. However, to definitively determine the indications and benefits of episiotomy, further studies involving larger patient groups are needed, along with consideration of its role in complex deliveries [50].

The use of perineal massage significantly reduced the need for episiotomies and shortened the second stage of labour. Therefore, this can be recommended as a safe, simple to apply, cost-effective technique, effective in reducing perineal trauma during labour [84]. However, its effectiveness in multiparous women is limited. According to a meta-analysis from 2020, perineal massage reduces the incidence of OASI by 64%, but these results are inconclusive due to the heterogeneity of the studies and potential publication bias [24]. A prospective randomised controlled trial conducted at a hospital in Braga from 2019 to 2023 demonstrated that the group using perineal massage and warm compresses had significantly higher rates of intact perineum and lower rates of second-degree tears and episiotomy compared to the control group. Furthermore, this group showed a reduced risk of anal sphincter injury and second-degree tears with episiotomy [85].

## 11. Ethical Aspects of Perinatal Birth Canal Injury

Perineal trauma, a common consequence of vaginal birth, can lead to severe, long-term physical and psychological issues for mothers [86,87]. The medical management of such trauma inherently involves significant ethical considerations.

Clinical practice concerning perineal trauma presents several ethical challenges. Achieving genuinely informed consent for procedures such as episiotomy can be particularly difficult during active labour; therefore, clear and early communication is crucial [88,89]. It is vital to remember that non-consensual procedures constitute a form of obstetric violence [87]. The debate surrounding routine versus clinically indicated interventions (e.g., episiotomy) also persists. Ethically, any intervention requires sound medical justification and must be individualised to the patient’s specific needs, as the routine overuse of procedures without clear indication can also be classified as obstetric violence [87]. Furthermore, the nature of staff communication and the level of support provided significantly impact the birthing person’s experience. Negative interactions can contribute to birth trauma, even if physical injury is minor [86,87]. It is also important to recognise that perineal injuries can result from various forms of obstetric violence, including neglect, abuse, discrimination, and non-consensual procedures [87]; such instances must be identified and actively counteracted. Consequently, building a trusting relationship with patients is fundamental for fostering cooperation and ensuring acceptance of necessary interventions [89].

In critical situations involving serious injury or high risk to the mother or child, the medical team must maintain composure and professionalism, ensuring that all actions taken are communicated clearly and respectfully [86]. While prioritizing the safety of both mother and child may necessitate rapid decision-making, every reasonable effort should still be made to inform the patient and obtain consent for procedures [88]. Effective communication and cooperation within the medical team are also paramount in these scenarios. Following birth, a thorough assessment and expert repair of any injuries are essential. If the birth experience is perceived as traumatic, the provision of post-trauma psychological support and opportunities for debriefing is an important aspect of care [86,89], particularly as a lack of respectful care has been shown to correlate with perceived birth trauma [86]. Finally, medical staff have an ongoing responsibility to update their clinical skills and engage in reflective ethical practice to minimise iatrogenic injuries and ensure patient-centred care.

## 12. Limitations and Future Directions of Research

There is a limited amount of research tracking long-term outcomes of female patients after injury, which makes it difficult to assess the long-term physical and psychological consequences. There is a lack of information on the impact of perineal injuries on quality of life and sexual functioning in the long term. Standardised diagnostic and therapeutic protocols are needed to unify guidelines and take into account differences in clinical practices between centres. An important question remains: how do perineal injuries affect the psychosocial functioning of patients, including mental health, interpersonal relationships, and occupational activity, and what psychosocial interventions should be undertaken?

## 13. Conclusions and Perspectives

Perineal lacerations during childbirth are influenced by numerous maternal, foetal, healthcare, and labour-related factors. Understanding and addressing these risk factors during prenatal and postpartum phases is essential in guiding preventive strategies. Episiotomy should be performed only when clinically indicated, rather than being used as a routine practice. Expectant mothers should be educated about potential risks and preventive measures, such as perineal massage, which has shown promise, especially in primiparous women, although further research is needed to confirm its effectiveness. Advanced classification systems and surgical techniques have improved diagnosis and treatment of perineal injuries, including severe cases like obstetric anal sphincter injuries (OASIs), yet challenges in accurate recognition and proper management remain significant. Properly matching the technique to the degree of perineal tear, as well as using appropriate surgical techniques and suturing methods, is essential for healing and minimizing complications following the injury. Ongoing efforts should focus on refining diagnostic tools, optimizing treatment protocols, establishing comprehensive training for healthcare providers, ensuring equitable access to skilled obstetric care, and exploring emerging technologies to enhance tissue healing. Given the significant physical and psychological effects of perineal trauma, which affect not only postpartum women but also their partners and potentially their bond with the newborn, a multifaceted and personalised therapeutic approach is crucial to improving maternal well-being, postpartum recovery, and overall quality of life.

## Figures and Tables

**Table 2:** Clinical issue that could be obtained during childbirth.

Clinical Issue	Description/ Mechanism	Risk Factors	Diagnosis	Symptoms/ Consequences	References
Episiotomy	Surgical incision to widen vaginal opening during delivery [50]	Forceps or spatula delivery, primiparity, foetal distress [51]	Clinical judgment and maternal or foetal factors during labour [50]	Easier delivery, may cause discomfort during recovery, affect mobility and sexual activity postpartum [50,52]	[50,51,52]
Obstetric fistula	Abnormal connection between vagina and bladder/rectum [53]	Prolonged, obstructed labour, limited access to medical care [54]	Dye tampon test, clinical examination [55] cystoscopy with ureteral assessment, CT, MRI [56]	Urinary/faecal incontinence, genital ulcers, social stigma, infertility, recurrent urinary tract infections, lack of sexual activity, and amenorrhea [57]	[53,54,55,56,57]
Uterine rupture	Complete tear of the uterine wall, (perimetrium, myometrium, and endometrium) [58]	Previous C-section, myomectomy, advanced maternal age, prior rupture, TOLAC, or later pregnancy [59]	Haemoglobin or haematocrit is the most important initial test for diagnosing uterine rupture, with imaging reserved for stable patients to rule out other causes of bleeding [58,60]	Haemorrhage, vaginal bleeding, abdominal pain, changes in contraction patterns, or a non-reassuring foetal heart rate tracing [58]	[58,59,60]
Dyspareunia	Ongoing or recurrent genital pain experienced before, during, or after sexual intercourse [61]	Type of delivery, episiotomy, breastfeeding, dyspareunia before or during pregnancy, number of previous births, and timing of postpartum sexual activity resumption [61]	Self-report by the patient [61]	Painful intercourse, reduced sexual function, distress [61]	[61]
PFD—Pelvic floor disorders	Pelvic floor dysfunction (PFD) encompasses a range of urologic, gynaecologic, and colorectal symptoms caused by abnormal pelvic muscle function or support, including conditions like pelvic organ prolapse (POP) [62,63]	Mechanical injuries such as anal sphincter tears, prolonged second stage of labour, instrumental delivery, multiparity, advanced maternal age, obesity, heavy physical labour, and genetic predisposition [64]	Urodynamics, cystoscopy. Anorectal manometry, balloon expulsion test, electromyography (EMG), endoanal ultrasonography, defecography dynamic MRI [63]	Limit women’s daily activities, reduce quality of life, and result in significant societal costs [62]	[62,63,64]
Baby blues	Mild, short-term depressive symptoms in the first days to weeks after childbirth [65]	Hormonal shifts, neural circuit dysfunctions in the reward system, delivery-related stress [66]	No formal diagnosis necessary [66]	Tearfulness, mood swings, irritability, anxiety, fatigue, and poor appetite [65]	[65,66]
Postpartum depression	Depressive disorder lasting weeks to months postpartum, impairing function [67]	Young maternal age, low education, poverty, unplanned pregnancy, lack of social support, poor family relationships, pregnancy or infant complications, formula feeding, and limited access to mental health care [64]	Screening tools (e.g., EPDS), psychiatric evaluation [65,68]	Difficulty bonding with the baby, persistent sadness and anxiety lasting at least two weeks, trouble concentrating [64,65]	[64,65,67,68]

## Data Availability

Not applicable.

## Abbreviations

The following abbreviations are used in this manuscript:

OASIs	Obstetric anal sphincter injuries
RCOG	Royal College of Obstetricians and Gynaecologists
VBAC	First vaginal birth after caesarean
FSI	Female Sexual Functioning Index
GRISS	Golombok Rust Inventory of Sexual Satisfaction
